# Diagnostic accuracy of a deep learning model using YOLOv5 for detecting developmental dysplasia of the hip on radiography images

**DOI:** 10.1038/s41598-023-33860-2

**Published:** 2023-04-24

**Authors:** Hiroki Den, Junichi Ito, Akatsuki Kokaze

**Affiliations:** 1 Department of Orthopaedic Surgery, National Rehabilitation Center for Children with Disabilities, 1-1-10 Komone, Itabashi-ku, Tokyo 173-0037 Japan; 2 Department of Hygiene, Public Health, and Preventative Medicine, Showa University School of Medicine, 1-5-8 Hatanodai, Shinagawa-ku, Tokyo 142-8555 Japan

**Keywords:** Orthopaedics, Radiography, Mathematics and computing, Medical imaging

## Abstract

Developmental dysplasia of the hip (DDH) is a cluster of hip development disorders and one of the most common hip diseases in infants. Hip radiography is a convenient diagnostic tool for DDH, but its diagnostic accuracy is dependent on the interpreter’s level of experience. The aim of this study was to develop a deep learning model for detecting DDH. Patients younger than 12 months who underwent hip radiography between June 2009 and November 2021 were selected. Using their radiography images, transfer learning was performed to develop a deep learning model using the “You Only Look Once” v5 (YOLOv5) and single shot multi-box detector (SSD). A total of 305 anteroposterior hip radiography images (205 normal and 100 DDH hip images) were collected. Of these, 30 normal and 17 DDH hip images were used as the test dataset. The sensitivity and the specificity of our best YOLOv5 model (YOLOv5l) were 0.94 (95% confidence interval [CI] 0.73–1.00) and 0.96 (95% CI 0.89–0.99), respectively. This model also outperformed the SSD model. This is the first study to establish a model for detecting DDH using YOLOv5. Our deep learning model provides good diagnostic performance for DDH. We believe our model is a useful diagnostic assistant tool.

## Introduction

Developmental dysplasia of the hip (DDH) is a cluster of hip developmental disorders, including dislocation, subluxation, and acetabular dysplasia. DDH is one of the most common hip diseases in infants. In our previous study, the incidence of DDH-related dislocations was 0.076% in Japan^1^. Early detection and treatment of DDH-related dislocations is highly effective, with a > 80% success rate^2–4^. However, treatment outcomes in patients diagnosed with DDH dislocation at the age of ≥ 1 year vary, suggesting that early detection and treatment are essential for good outcomes. In our previous study, we reported that the rate of late diagnosis (diagnosed ≥ 1 year) was 10–12% in Japan, which is concerning^1^.

Hip radiography and ultrasonography are widely used screening tools for diagnosing DDH. Hip ultrasonography is an accurate modality, but it requires a certain level of experience to achieve acceptable performance^5^. Therefore, it may be difficult to popularize the hip ultrasonography technique for physicians who conduct hip screening in infants. Hip radiography is a convenient diagnostic tool since it is available in most hospitals and clinics. However, the diagnostic accuracy of hip radiography for DDH varies according to the interpreter’s experience^6^. DDH is a relatively rare disease; in fact, a Japanese study revealed that 32% of orthopedic doctors were yet to encounter a patient with DDH in their career as at the time of the study^1^. A tool that can help inexperienced doctors diagnose DDH may be necessary and can reduce the percentage of late diagnoses.

Deep learning technology has rapidly progressed and is widely used to detect or classify objects on images in many fields, such as face recognition. Recent studies have shown that deep learning techniques can also be applied to radiographic images^7–11^.

To our knowledge, studies that have applied deep learning techniques to detect DDH using hip radiography images are limited^12,13^. In this study, the “You Only Look Once” v5 (YOLOv5) method, which is one of the most widely used object detection methods, was used^14^. Object detection methods include one- and two-stage approaches. The one-stage object detection methods mainly used are the YOLO series and single shot multi-box detector (SSD), while the faster R-CNN is a widely adopted two-stage object detection method^15,16^. The one-stage methods can complete both classification and position detection tasks simultaneously, and are therefore popular because of their speed and accuracy^17,18^. The YOLO series is particularly renowned for its flexible structure, and many researchers have conducted object detection using improved algorithms based on the YOLO algorithm. YOLOv5 was developed in 2020, and it has been used in various applications to detect traffic signals, people, parking meters, and animals^19–21^.

The aim of this study was to use the YOLOv5 and SSD to develop deep learning models that can be used to distinguish between a normal and DDH hip, using hip radiography images. We also sought to validate the diagnostic performances of the developed models.

## Methods

This retrospective study was approved by the Institutional Review Board of the National Rehabilitation Center for Children with Disabilities (approval no. 2014-17), and all methods were carried out in accordance with their guidelines and regulations. Written informed consent was obtained from the parents or the legal guardians of all children.

### Data collection

Patients younger than 12 months who were suspected to have DDH-related hip dislocation or hip subluxation and who had undergone anteroposterior (AP) view hip radiography between June 2009 and November 2021 were selected retrospectively. Radiography images were evaluated for appropriate standard AP view of the hip. The rotation quotients were obtained by dividing the transverse diameter of the obturator foramen on the right side by that of the left side. Radiography images with rotation quotients ranging from 0.5 to 2.0 were considered appropriate, while those taken in an inappropriate position were excluded^22,23^. For patients who underwent several hip radiographs, the oldest image in the time series was selected, because the AP view hip radiographs were taken just once by the age of 12 months, in most patients. All patients were screened by the same experienced pediatric hip specialist for DDH. Patients were diagnosed with DDH based on physical examination findings, AP view hip radiography images, and hip ultrasonography images, if necessary. A positive DDH diagnosis using hip radiography images was based on the following criteria: (a) lateralization of the epiphyseal ossification center, (b) interruption of the Shenton line^24^, (c) widened teardrop distance compared to that of the other side^25^, (d) delayed femoral head ossification compared to that of the other side, (e) high acetabular index (> 30), and (f) dulled edge of the acetabulum. The Graf method was used for hip ultrasonography^26^. The International Hip Dysplasia Institute (IHDI) classification was used to quantify DDH severity because the classification does not rely on the presence of the ossification center of the femoral head and it can be applied to patients of all ages^27^.

Patients with DDH of IHDI grade 2 or worse and/or type 2c or worse Graf classification based on the hip ultrasonography images were generally considered to have DDH, as they require close monitoring^26^.

### Data preparation

The original images were 1430 × 1140 pixels in size. These images were changed into a square shape (1430 × 1430 pixels) by adding black regions to the top and bottom. Then, the images were resized to 864 × 864 pixels. Approximately 15% of each normal and DDH images were randomly but equally distributed between the validation and test datasets, considering the equality of DDH severity based on IHDI classification. For the training dataset, all images were augmented by flipping them horizontally. In addition, to avoid overfitting, the DDH images were augmented by 10° and − 10° rotations.

### Image annotation

**Figure 1:**
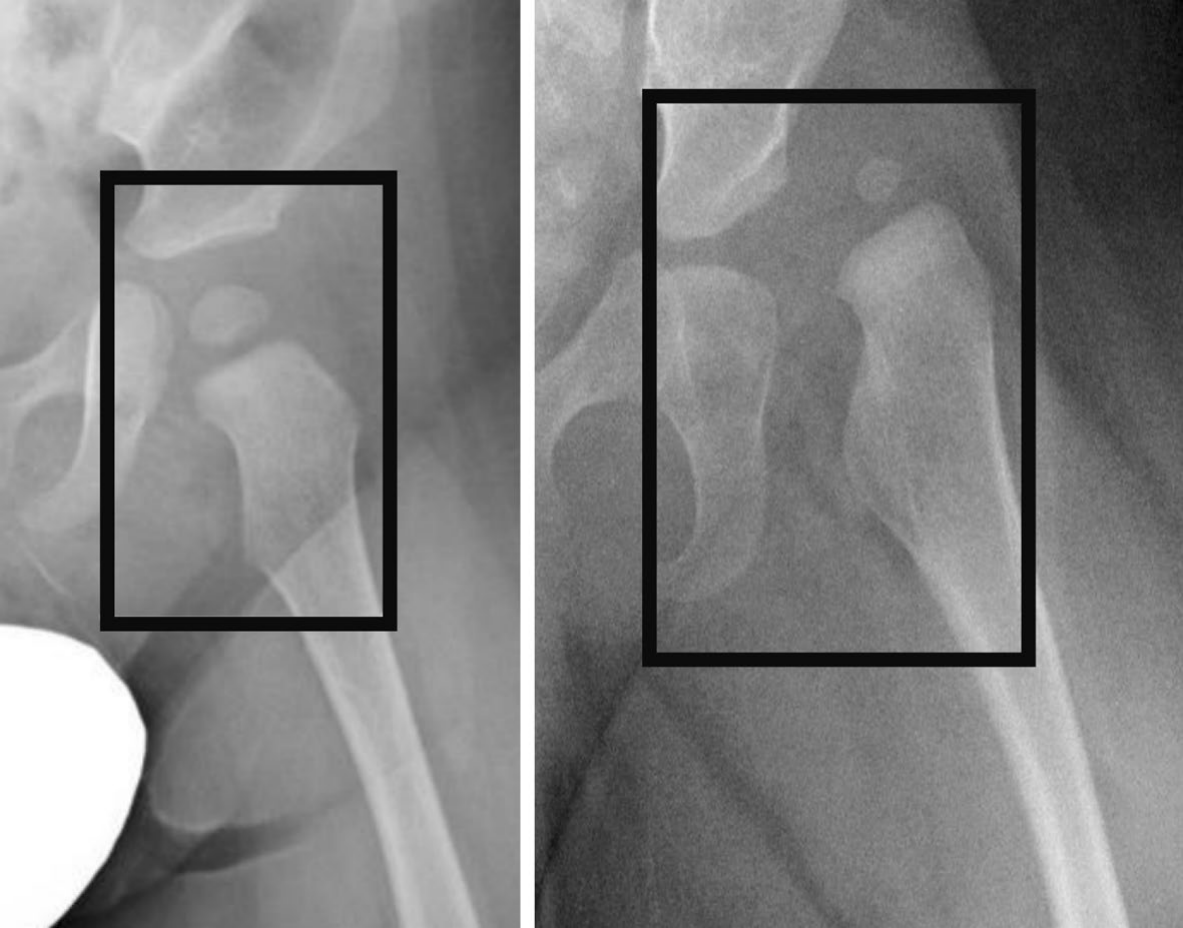
Annotation examples. Examples of bounding boxes for normal (left image) and DDH (right image) hips. *DDH* Developmental dysplasia of the hip.

Image annotation was conducted using LablImg version 1.8.1 with reference to the diagnosis made by the experienced pediatric hip specialist (J.I.)^28^. Object bounding boxes were drawn to encompass the entire hip joint, based on the following criteria: (a) the inner boundary is drawn in anatomical regions deeper than the deepest region of the acetabulum, (b) the outer boundary is drawn to include the greater trochanter, (c) the upper boundary is drawn to include the acetabulum and the ossification center of the femoral head, and (d) the lower boundary is drawn to include the lesser trochanter (Fig. 1). Image annotations for the normal and DDH hips were performed in similar manner, based on the criteria above. Normal hips are labeled as “Normal” and DDH hips as “DDH”. Following the image annotation process, all images were reviewed by the senior orthopedic doctor (J.I.).

### YOLOv5 algorithm

Transfer learning, a technique in which a well-trained model from a large dataset is used for applications of interest with a small dataset, was performed using YOLOv5^29^. Therefore, transfer learning can reduce the requirement of large datasets. YOLOv5 contains four different models: YOLOv5s, YOLOv5m, YOLOv5l, and YOLOv5x. The main difference between these models is the amount of their feature extraction modules. YOLOv5s has the smallest size of modules and amount of module parameters, and YOLOv5x has the largest size of modules and amount of module parameters^19^. All four models were utilized for the present study and results were compared. For transfer learning, the first 10 layers of the YOLOv5 models were frozen in place, and the rest of the layers were retrained using our new datasets. YOLOv5 is composed of three types of loss functions: box loss, object loss, and class loss.

The box loss represents the localization loss of the positive predictions, and used complete IOU (CIOU). The object loss represents the CIOU loss of the detection and true boxes, defined as binary cross entropy loss. The class loss represents the classification loss of the positive predictions, defined as a binary cross-entropy loss^30–32^. Our models used the stochastic gradient descent (SGD) as an optimizer, with a learning rate of 0.01, mini-batch size of 32, and 100 epochs, for the training.

### SSD algorithm

The backbone network of SSD adopts the Visual Geometry Group (VGG) 16 feature extraction network, which is widely used in feature extraction, and the extra feature layers added after the backbone network is used to achieve the multi-scale detections^15,33^. We also applied transfer learning by loading the pre-trained VGG16 model^34^. The loss function of the SSD model consists of the localization and confidence losses. The overall loss function is the weighted sum of these two^15^. The localization loss is a smooth L1 loss between the predicted box and the ground truth box parameters, while the confidence loss is the softmax loss over multiple classes confidences^15^. Images were transformed to 300 × 300 pixels in size and the model used SGD as the optimizer with a learning rate of 0.01, mini-batch size of 32, and 200 epochs for the training. The same training and validation datasets used for YOLOv5 models were used.

**Figure 2:**
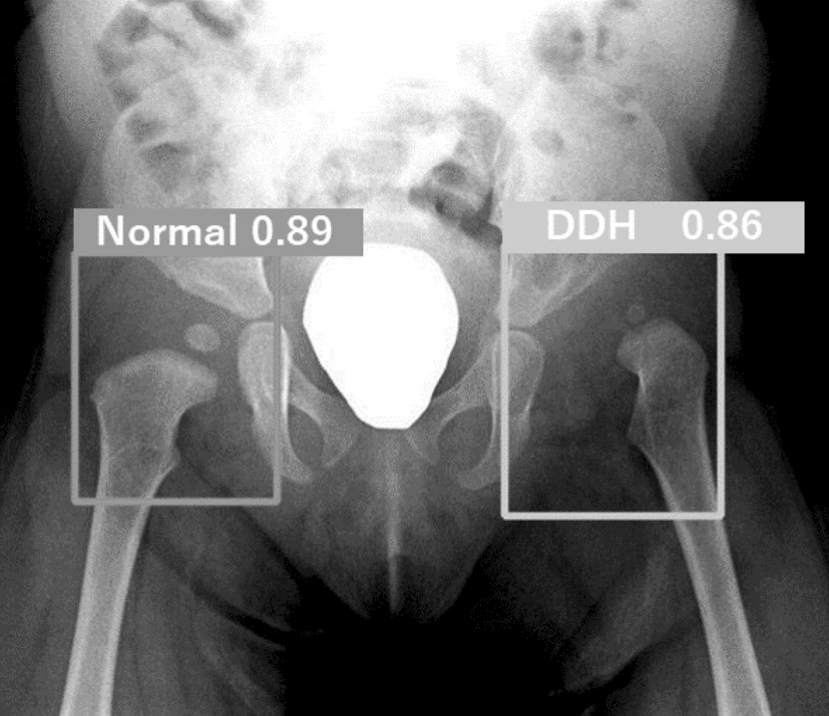
Example of a test image outcome. The right hip was labeled “Normal” and the left hip was labeled “DDH”, with class-specific confidence scores. Both hips were correctly labeled. *DDH* Developmental dysplasia of the hip.

The analyses were performed using Python 3.7.12 (Python Software Foundation, Wilmington, DA, U.S.). Consequently, the trained models could detect hips in AP view radiography images and label them as either “Normal” or “DDH”, with class-specific confidence scores (Fig. 2).

The confidence scores indicate how the level of confidence that the box contains an object and how well the predicted box fitted the object. Class-specific confidence scores were calculated by multiplying the class probabilities and individual box confidence scores. These scores indicate the probability of a correct class labeling and how well the predicted box fitted the labeled class^20,30^.

**Figure 3:**
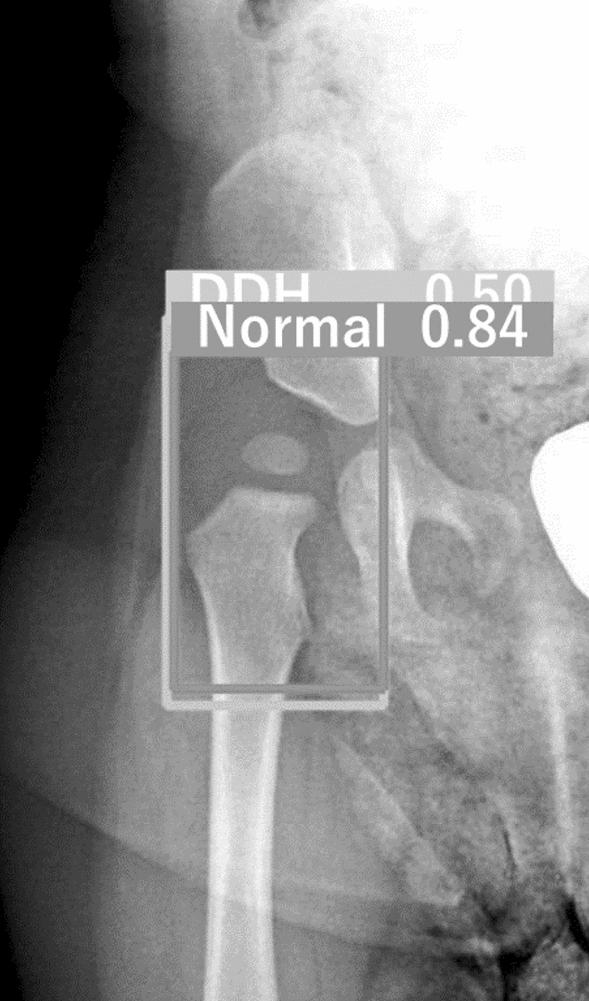
Example of double labeling. The right hip was labeled as both “Normal” and “DDH”. *DDH* Developmental dysplasia of the hip.

The test dataset was evaluated using the trained models, at a 0.5 confidence score threshold. If both “Normal” and “DDH” were labeled on the same hip, the hip evaluation was considered invalid (Fig. 3). Accuracy, sensitivity, specificity, positive predictive value, and negative predictive value were calculated for each trained model.

## Results

**Figure 4:**
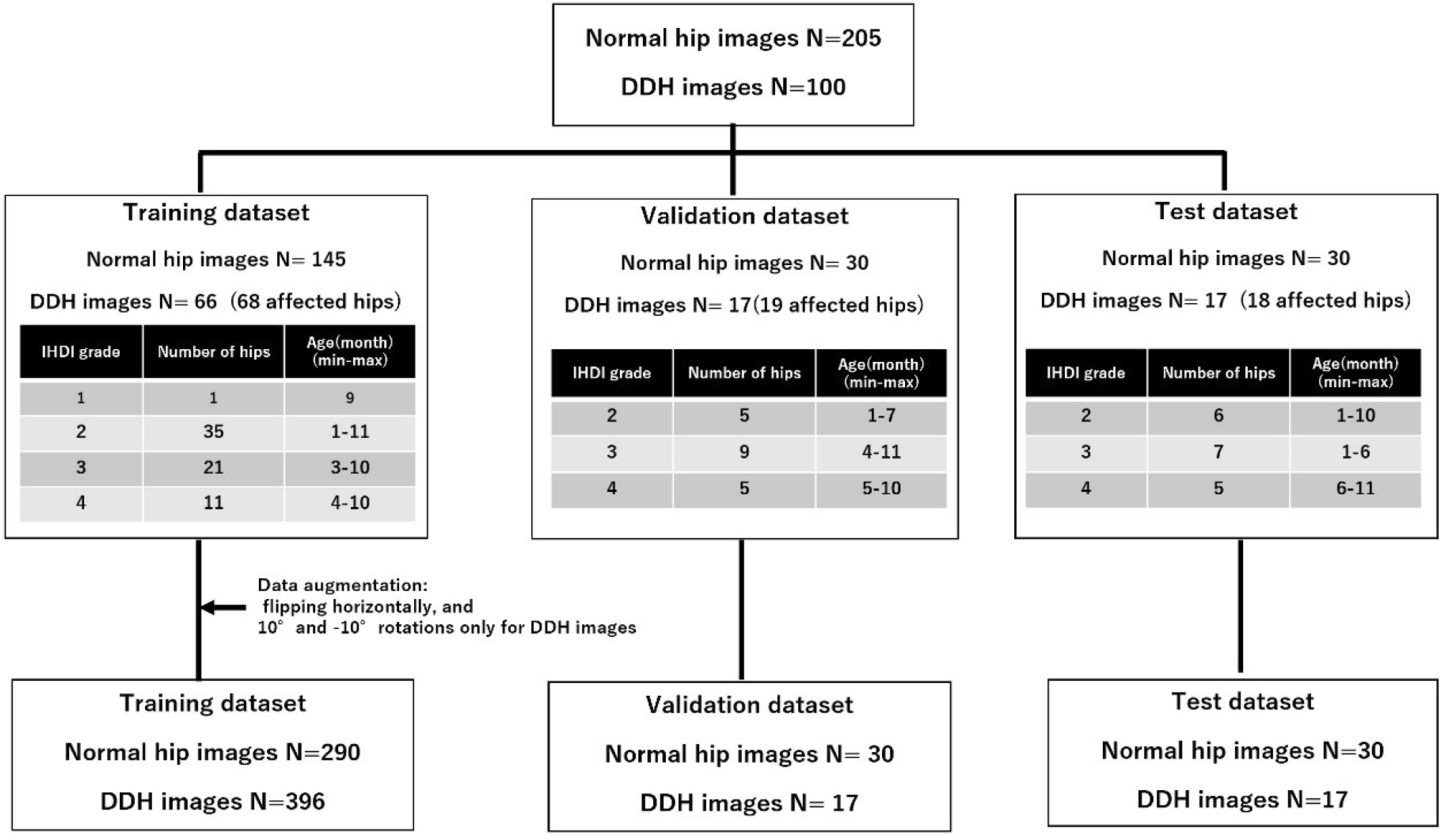
Flowchart of data preparation. DDH: developmental dysplasia of the hip.

Between June 2009 and November 2021, 305 hip AP X-ray images (205 normal hip images and 100 DDH hip images) were collected. Of the normal hip group, 171 patients (83.4%) underwent hip ultrasonography, while in the DDH group, 87 patients (87%) received hip ultrasonography to confirm the diagnosis. In particular, all IHDI grade 2 patients, who were under 6 months of age, underwent hip ultrasonography. As a result, 396 DDH and 290 normal images were utilized for training, while 30 normal and 17 DDH hip images were used for testing (Fig. 4). No age bias was observed between learning, validation, and test datasets.

**Figure 5:**
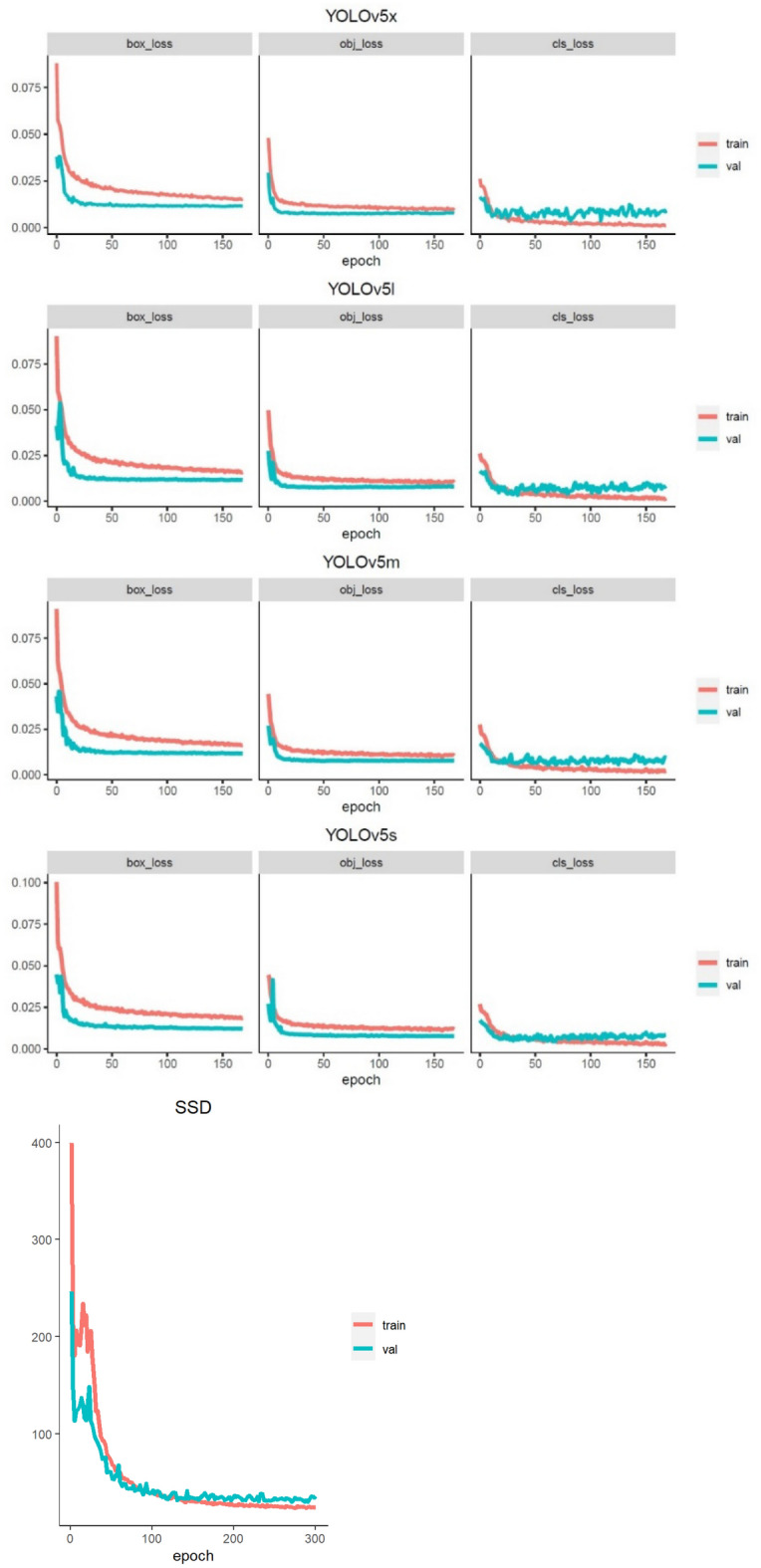
Training and validation curves for the YOLO and SSD models. Box_loss: box loss. Obj_loss: object loss. Cls_loss: class loss. *SSD* Single shot multi-box detector, *YOLO* “You Only Look Once”.

The basic characteristics of the datasets are shown in Table 1. One case had an IHDI grade of 1 in the DDH group, but the patient’s Graf classification was type 2c. Therefore, this case was categorized as DDH. The training and validation loss curves are shown in Fig. 5.

**Table 1:** Basic characteristics.

		DDH group N = 100	Normal group N = 205
Girls, n (%)		88 (88)	152 (74.1)
Age (month) mean (min–max)		6.1 (1–12)	6.0 (0–12)
Affected side	Left	69	
	Right	26	
	Bilateral	5	
IHDI grade	1	1	
	2	46	
	3	37	
	4	21	

*DDH* Developmental dysplasia of the hip, *IDHI* International Hip Dysplasia Institute.

Among the four YOLO models, four hips were labeled as both “Normal” and “DDH”; one hip in the YOLOv5x model and three hips in the YOLOv5s model. There was no double labeling in the other two models, as well as in the SSD model. Table 2 shows the diagnostic performance of each model for the test dataset.

**Table 2:** Diagnostic performances of each model using the test dataset.

	Accuracy (95% CI)	Sensitivity (95% CI)	Specificity (95% CI)	PPV (95% CI)	NPV (95% CI)
v5x	0.96 (0.89–0.99)	0.83 (0.59–0.96)	0.99 (0.93–1.00)	0.94 (0.70–1.00)	0.96 (0.89–0.99)
v5l	0.96 (0.89–0.99)	0.94 (0.73–1.00)	0.96 (0.89–0.99)	0.85 (0.62–0.97)	0.99 (0.93–1.00)
v5m	0.96 (0.89–0.99)	0.89 (0.65–0.99)	0.97 (0.91–1.00)	0.89 (0.65–0.99)	0.97 (0.91–1.00)
v5s	0.94 (0.87–0.98)	0.94 (0.73–1.00)	0.93 (0.85–0.98)	0.77 (0.55–0.92)	0.99 (0.93–1.00)
SSD	0.96 (0.89–0.99)	0.89 (0.65–0.99)	0.97 (0.91–1.00)	0.89 (0.65–0.99)	0.97 (0.91–1.00)

*CI* Confidence interval, *PPV* Positive predictive value, *NPV* Negative predictive value, *SSD* Single shot multi-box detector.

In terms of accuracy, all the models except YOLOv5s showed similar results.

For false negatives, the YOLOv5s and YOLOv5l models mislabeled only one hip, and the same hip was found to have been mislabeled by the models.

All severe DDH cases (IHDI grades 3 and 4) were correctly labeled as “DDH” in all YOLO models, but one IHDI grade 3 case was mislabeled in the SSD model. The labeling of mild DDH cases (IHDI grade 2) varied, and no model labeled all IHDI grade 2 cases correctly (Table 3).

**Table 3:** DDH hips correctly labeled “DDH”, according to IHDI grade.

IHDI grade	Total	N (%)				
		YOLOv5x	YOLOv5l	YOLOv5m	YOLOv5s	SSD
4	5 hips	5 (100)	5 (100)	5 (100)	5 (100)	5 (100)
3	7 hips	7 (100)	7 (100)	7 (100)	7 (100)	6 (85.7)
2	6 hips	3 (50.0)	5 (83.3)	4 (66.7)	5 (83.3)	5 (83.3)

*DDH* Developmental dysplasia of the hip, *IDHI* International Hip Dysplasia Institute, *SSD* Single shot multi-box detector, *YOLO* “You Only Look Once”.

## Discussion

We developed deep learning models for detecting DDH using hip radiography images in the AP view. To the best of our knowledge, this is the first study to achieve this using YOLOv5 and SSD. In addition, using the transfer learning technique, a good model could be constructed with a relatively small dataset. The benefit of using an object detection model rather than a classification model is the ability of the object detection model to evaluate both hips simultaneously without image processing, and that the image outcomes are easy to understand.

The disadvantage of YOLOv5, in general, is that it is not optimized to detect small objects^35^. Because the hips are large enough on hip radiography images, this drawback did not seem to affect our results. Studies that used YOLOv5 models have been reported in various medical fields^36^. One study used YOLOv5 models to detect brain abnormalities, and another study sought to detect lumbar spine deformities using YOLOv5 models^37,38^. As a confirmatory diagnostic tool, hip ultrasonography is probably the best modality in the hands of a well-trained operator. A study reported that the sensitivity and specificity of hip ultrasonography for detecting DDH were 93% and 97%, respectively^5^. The diagnostic performances of our trained models were comparable, but the diagnostic performance of hip ultrasonography would be better than that in the current study if the operator is well-trained.

The strength of a deep learning model compared to hip ultrasonography screening is that it can be applied in clinics that do not have access to a pediatric hip specialist. By using a deep learning model, a general practitioner with insufficient expertise in orthopedics might be able to diagnose DDH. Combining telemedicine with deep learning technology may decrease the rate of late DDH diagnosis. An autonomous artificial intelligence-based diagnosis system has already been applied in the field of medicine^39^.

A study reported the sensitivity and specificity of the AP view hip radiography images, reviewed by radiologists for the detection of DDH. The Sensitivity and specificity of 96.0% and 89.0%, respectively, were indicated for a radiologist with a 5-year radiology experience, including pediatric radiology, while 84.0% and 85.8% were reported for one with a 3-year radiology experience, but without any pediatric radiology experience^13^. These results indicate that our deep learning model can be a useful screening tool for physicians who do not have sufficient experience regarding DDH.

High sensitivity is desirable for screening tools. Among our four trained YOLO models, the YOLOv5l and YOLOv5s models had the highest sensitivity (0.94). In contrast, YOLOv5x had the highest specificity (0.99) while YOLOv5s had the lowest specificity (0.93). Overall, of the four YOLO models, YOLOv5l was considered the best screening tool because of its high sensitivity and specificity. The diagnostic performance of the SSD model is equivalent to that of the YOLOv5m model and considering that the one IHDI grade 3 DDH case was mislabeled, the YOLOv5l model also seems to be superior to the SSD model. One study reported the comparison of traffic sign recognition performance between YOLOv5 and SSD using their own dataset, and YOLOv5 outperformed SSD in terms of the mean average precision (mAP)^40^. Another study reported the comparison of pavement crack detection performance between YOLOv4 and SSD, and the YOLOv4 also outperformed SSD in terms of mAP^17^. These results are similar to ours.

There were four hips labeled as both “Normal” and “DDH” in the YOLOv5x and the YOLOv5s models. As a screening tool, high sensitivity is crucial. Therefore, in practice, these double-labeled cases should be treated as “positive”, and further examination should be performed.

**Figure 6:**
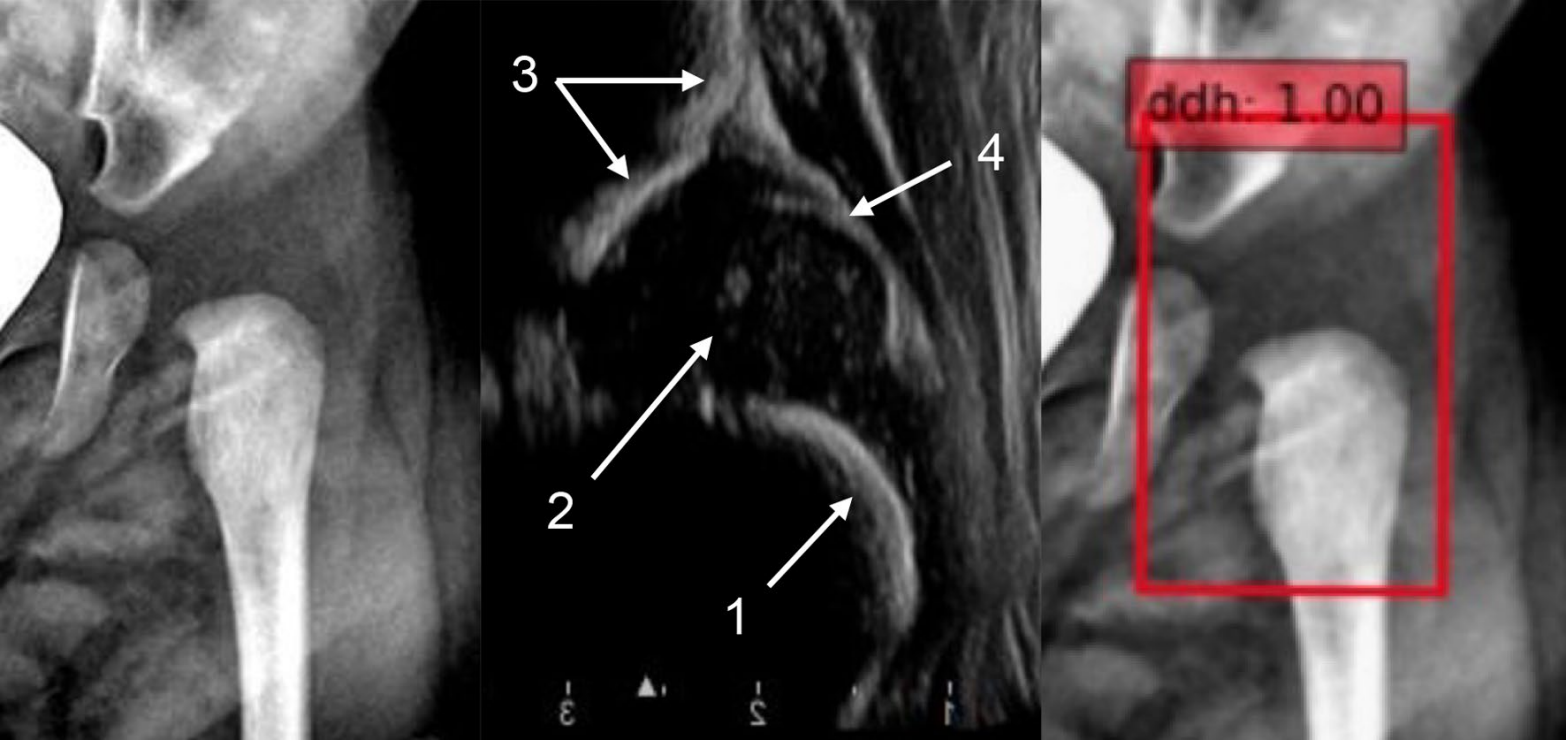
Hip anteroposterior radiography image (left), hip ultrasonography image of the same hip (middle), and the SSD model image output (right). The patient was a 1-month-old boy. IHDI grade 2, Graf type D. Anatomical interpretation of the ultrasonography image: 1, bony part of the femoral neck. 2, cartilaginous femoral head. 3, bony part of acetabular roof. 4, acetabular labrum. *IDHI* International Hip Dysplasia Institute, *SSD* Single shot multi-box detector.

**Figure 7:**
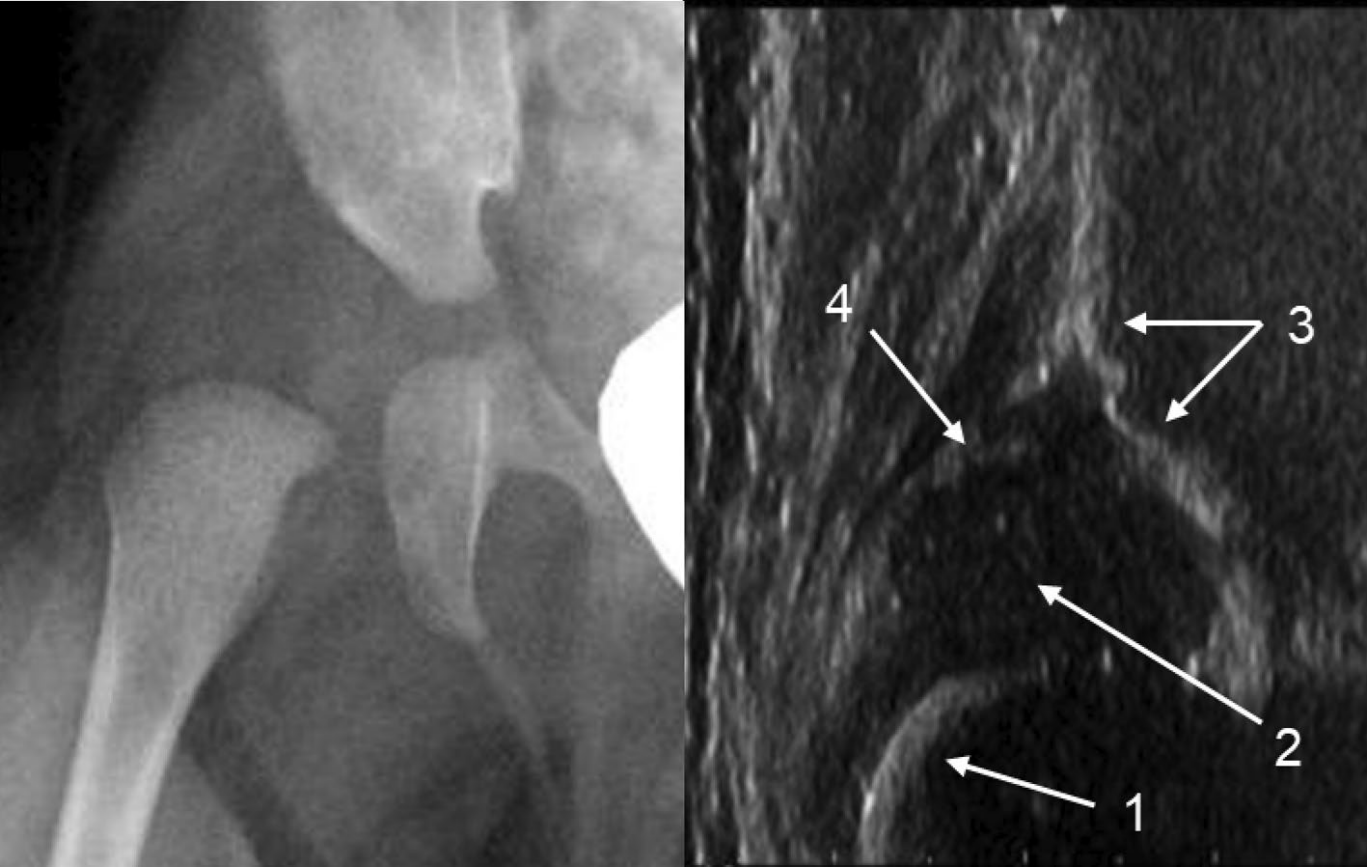
Hip anteroposterior radiography image (left) and hip ultrasonography image of the same hip (right). The patient was a 2-month-old girl. IHDI grade 2, Graf type D. Anatomical interpretation of the ultrasonography image: 1, bony part of the femoral neck. 2, cartilaginous femoral head. 3, bony part of acetabular roof. 4, acetabular labrum. *IDHI* International Hip Dysplasia Institute.

There was only one false-negative diagnosis for the YOLOv5l model. Furthermore, all four YOLO models mislabeled the same case, which only the SSD model correctly labeled (Fig. 6). Another DDH case mislabeled by the YOLOv5x, YOLOv5m, and SSD models is shown in Fig. 7. Both patients were less than 2 months old, and hip epiphyseal ossification centers could not be detected in the hip radiography images.

**Figure 8:**
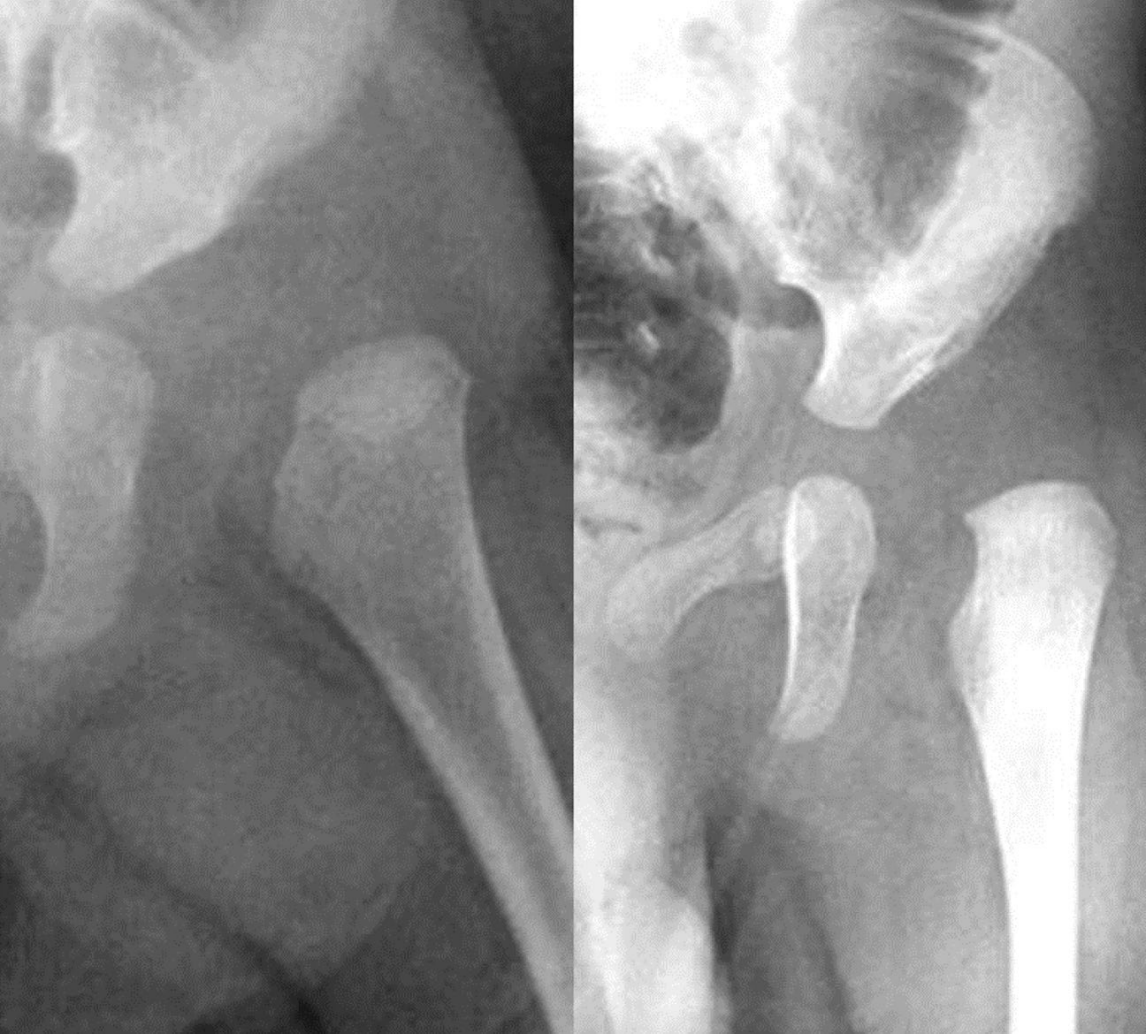
A 3-month-old girl with IHDI grade 3 DDH (left) and a 1-month-old girl with IHIDI grade 3 DDH (right). Both hips were correctly labeled as DDH, although epiphyseal ossification centers did not appear. *DDH* Developmental dysplasia of the hip, *IDHI* International Hip Dysplasia Institute.

Compared to our trained models, hip ultrasonography may be a superior tool for evaluating neonatal (before the appearance of the epiphyseal ossification center) and mild cases of DDH. However, our four YOLO-trained models could correctly detect DDH in all severe DDH (IHDI grades 3 and 4) cases. Furthermore, in two patients aged less than 4 months in whom the epiphyseal ossification centers could not be detected radiographically, DDH was correctly detected (Fig. 8).

All trained models did not display satisfactory diagnostic accuracy for IHDI grade 2 DDH. However, as mentioned above, there was only one false-negative diagnosis for the YOLOv5l model, whereas, the SSD model could correctly label the same case. If hips labeled as “DDH” in either YOLOv5l or SSD model had been treated as “DDH”, the sensitivity of the test dataset would have achieved 1.00 while the specificity was still acceptable (0.93 [95% CI 0.85–0.98]). As such, our trained models could be a useful diagnostic assistant tool for any IHDI grade of DDH.

Even though YOLOv5x had the highest number of parameters, the YOLOv5l model outperformed theYOLOv5x model in terms of sensitivity. Similar results were found in another study that developed models to detect personal protective equipment using YOLOv5^21^. In that study, the YOLOv5m model outperformed the YOLOv5l and YOLOv5x models in terms of precision and recall. Class-specific confidence scores were almost always high whether the hips were correctly labeled or not. For example, in the YOLOv5l model, all confidence scores were greater than 0.7, and 97% of the confidence scores were greater than 0.8. In addition, all three hips with confidence scores less than 0.8 were labeled correctly. Therefore, in our models, the class-specific confidence scores did not seem to play an important role in DDH diagnosis. Because the difference between hips with or without DDH was subtle, confidence scores were probably high in most cases. We found a few similar studies. One study reported the diagnostic performance of a convolutional neural network deep learning algorithm for detecting DDH. The sensitivity and specificity of the algorithm were 0.94 and 0.99, respectively^13^. In another study, a deep learning algorithm was applied to measure the acetabular index and center–edge angle and provide IHDI classification, and IHDI classification accuracies ranged from 0.86 to 0.95^12^. These study results are comparable to our results.

This study has several limitations. First, it was a single-center study with a relatively small sample size of only 100 DDH hip images were collected. Using more data would improve the deep learning model, and further studies with larger datasets are needed to evaluate different ages and grades of DDH more accurately. Second, in this study, we only developed the models using the one-stage object detectors, and studies using the two-stage object detectors such as Faster R-CNN should also be considered.

Third, the same physician made all diagnoses, which may have introduced bias. However, the diagnoses were based not only on radiographic findings but also on physical examination and hip ultrasonography findings, suggesting sufficient diagnostic accuracy. Finally, patients who required careful observation were included in the DDH group in this study, which may be considered an overdiagnosis. We believe that with a screening tool, overdiagnosis is better than underdiagnosis.

In conclusion, our deep learning models using YOLOv5 provided accurate diagnostic performances for DDH. We believe our model is a useful diagnostic assistant tool.

## Data Availability

The datasets analyzed in this study are available from the corresponding author upon a reasonable request.
